# Antihyperglycemic and antidyslipidemic activity of *Musa paradisiaca*‐based diet in alloxan‐induced diabetic rats

**DOI:** 10.1002/fsn3.538

**Published:** 2017-11-20

**Authors:** Basiru O. Ajiboye, Hussein O. B. Oloyede, Musa O. Salawu

**Affiliations:** ^1^ Department of Biochemistry Faculty of Life Sciences University of Ilorin Ilorin Nigeria; ^2^ Department of Chemical Sciences College of Sciences Afe Babalola University Ado‐Ekiti Nigeria

**Keywords:** dyslipidemia, hyperglycemia, *Musa paradisiaca*, unripe

## Abstract

This study was aimed at investigating the antihyperglycemic and antidyslipidemic activity of *Musa paradisiaca*‐based diets in alloxan‐induced diabetic mellitus rats. Diabetes was induced by a single intraperitoneal injection of alloxan (150 mg/kg b.w) in 48 randomly selected rats. The rats were randomly grouped into four as follows: normal rats fed *Dioscorea rotundata*‐based diet, diabetic control rats fed *D. rotundata*‐based diet, diabetic rats fed *D. rotundata*‐based diet and administered metformin (14.2 mg/kg body weight) orally per day, and diabetic rats fed *M. paradisiaca*‐based diet. Body weight and fasting blood glucose level were monitored, on 28th days the rats were sacrificed, liver was excised. Thereafter, the hyperglycemic and dyslipidemic statii of the induced diabetic animals were determined. The *M. paradisiaca*‐based diet significantly (*p *<* *.05) reversed the levels of fasting blood glucose, with significant (*p *<* *.05) increase in insulin and glycogen concentrations. The diet also increased the activity of hexokinase with significant reduction (*p *<* *.05) in glucose‐6‐phosphatase and fructose‐1‐6‐diphosphatase activities. *M. paradisiaca*‐based diet demonstrated significant reduction (*p *<* *.05) in cholesterol, triacylglycerol (TG), very low‐density lipoprotein (VLDL), low‐density lipoprotein (LDL), and significant increase (*p *<* *.05) in high‐density lipoprotein (HDL) compared with those of diabetic control group. Also, *M. paradisiaca*‐based diet significantly (*p *<* *.05) reversed the activities of aspartate aminotransferase and alanine aminotransferase when compared with diabetic control animals. The consumption of this diet may be useful in ameliorating hyperglycemia and dyslipidemia in diabetes mellitus patients.

## 1 INTRODUCTION

Diabetes mellitus is a metabolic disorder that affects people of various age groups and from all walks of life. There is an estimate of more than 382 million people worldwide suffering from diabetes mellitus (International Diabetes Federation, 2011). Bisht & Sisodia (2010) reported that hyperglycemia and dyslipidemia are one of the main complications of diabetes mellitus. Hyperglycemia in diabetes mellitus state may be attributed to deficiency in insulin concentration, which triggers increase in blood glucose concentration and affecting activities of some carbohydrate metabolizing enzymes (glucose‐6‐phosphatase and hexokinase among others). Dyslipidemia is a phenomenon of altered levels of lipid, manifesting as low levels of high‐density lipoprotein‐cholesterol (HDL‐cholesterol) and high levels of low‐density lipoprotein‐cholesterol (LDL‐cholesterol) and triglycerides (Bisht & Sisodia, 2010). Management of diabetes mellitus without side effects is still a challenge because presently available drugs for diabetes have one or more adverse effects (such as hypoglycemia, gastrointestinal disorders, kidney complications, skin rash, dizziness, etc.) (Bohannon, 2002). This has led to the search for new drugs, recently, herbal remedies have been gaining importance in this regards, but they are characterized with few side effects (Rao, Sudarshan, Rajsekher, Nagaraju, & Rao, 2003).

Johnson, Isaac, Michael, Akintayo, & Samuel (2013) suggested that dietary intervention is the simplest and cheapest form of diabetic mellitus treatment. This is clinically recommended as the primary therapy in diabetes mellitus. Therefore, diabetic mellitus patients need dietary formulations that are easily available and affordable in their environment (Atangwho, Agiang, Alozie, & Ani, 2012; Johnson, Isaac, Michael, Akintayo, & Samuel, 2013). An example of such food is mature unripe *Musa paradisiaca* (unripe plantain). Indeed, the use of this food as component of diet has been acclaimed in Nigeria to be effective in the management of diabetes mellitus.

*Paradisiaca* (plantain) belongs to the family of Musacace and is cultivated in many tropics and subtropical countries of the world. These include Southern United states, Central America, Africa (e.g., Nigeria, Cameroun, etc.) (Gawel, 1995). *Paradisiaca* ranks third after yam and cassava for sustainability in Nigeria (Akomolafe & Aborisade 2007; Ayodele & Godwin, 2010). It is usually cultivated for its carbohydrate content and can be consumed as an unripe fruit or when ripe (Ahenkora, Kye, Marfo, & Banful, 1997). Adegboyega (2006) reported that mature unripe *Musa paradisiaca* is very rich in iron, potassium, vitamin A, ascorbic acid, and protein and has antioxidant potential than ripe ones. Therefore, this study is aimed at investigating the antihyperglycemic and antidyslipidemic activities of unripe *M. paradisiaca‐based* diet in alloxan‐induced diabetic rats.

## 2 MATERIALS AND METHODS

### 2.1 Samples collection and identifications

Mature unripe *M. paradisiaca* (unripe plantain) was purchased from Esekayah Village in Oriade Local Government, Osun State, Nigeria. *Dioscorea rotundata* (white yam) was purchased from Oja Oba Market in Ilorin, Kwara State, Nigeria. These were authenticated in the Department of Plant Biology, University of Ilorin, Ilorin, Nigeria and were, respectively, assigned voucher numbers UIH001/1186 and UIH004/482.

### 2.2 Ingredients used for diets composition

Rice husk and soybean were purchased from Oja Oba market in Ilorin, Kwara State, Nigeria. Soybean oil was a product of Sunola Refined Soybeans, Kewalram Nigeria Limited, Nigeria. Vitamin/mineral mix was a product of Rofat Feed Nigeria Limited, opposite Government Day Secondary School, Maraba Road, Ilorin, Kwara State, Nigeria.

### 2.3 Drug, chemicals, and assay kits

Metformin used in this study was a product of Merck Sante, France. Alloxan monohydrate and all chemicals used were products of Sigma Chemical Company, St Louis Mo USA ,while all assay kits used were products of Randox Laboratories Co‐Artrim, United Kingdom.

### 2.4 Processing of the plants materials

Unripe *M. paradisiaca* and *D. rotundata* (this was used as a control diet due to its favorite consumption in the Western part of Nigeria, where the experiment was carried out) were peeled, sliced, and oven dried at 60°C (for 72 hr). Each sample was thereafter milled separately with local grinding machine and kept separately.

### 2.5 Proximate composition determination

The proximate analyses of the compounded diets were determined using AOAC (1990) methods. Arabinose, fructose, and soluble and insoluble dietary fiber were determined using AOAC (2001) methods. Minerals estimation was carried out using AOAC (1980), while the determination of vitamins and amino and fatty acids was carried out using AOAC (2005) method.

### 2.6 Preparation of aqueous extracts for the compounded diets

Ten grams milled of each of the compounded diets was extracted in 100 ml of distilled water (for 24 hr). This was then filtered using Whatmam filter paper. Thereafter, the filtrate was freeze dried as described by Oboh, Puntel, & Rocha (2007), redissolved in distilled water, and kept for subsequent analyses.

### 2.7 Determination of phenolic contents and *in vitro* antioxidants of the compounded diets

Total phenol and total flavonoids were determined using Wolfe, Wu, & Liu (2003) and Bao, Cai, Sun, Wang, & Corke (2005), respectively. Ferric reducing power (FRAP), nitric oxide, iron chelation, 2,2 diphenyl‐1‐picrylhydrazyl, and hydroxyl radical were determined as described, respectively, by Pulido, Bravo, & Saura‐Calixto (2000), Jagetia & Baliga (2004), Minotti & Aust (2000), Liyana‐ Pathiranan & Shahidi (2005), and Halliwell & Gutteridge (1989).

### 2.8 Laboratory animals

A total of 48 albino rats (*Rattus norvegicus*) comprising of male and female with an average weight of 150 ± 20 g (2 ½ to 3 months old) were used for the experiment. They were obtained from the Animal House of the Department of Biochemistry, University of Ilorin, Nigeria. The animals were kept in a well‐ventilated house with free access to food and drinking water during the entire experimental period. The protocol used in this study was approved by the University of Ilorin Ethical Committee with ethical number BCH/SCI/029.

### 2.9 Induction of diabetes

Alloxan monohydrate of 150 mg/kg bodyweight (Osinubi, Ajayi, & Adesiyun, 2006) was administered intraperitoneally to 36 albino rats to induce the diabetes. The fasting blood glucose levels of these rats were previously determined after 12 hr of fasting. After 48 hr of induction, the tail arteries of these animals were punctured to collect the blood and used in determining their blood glucose levels using Accu‐check active glucometer. Rats with fasting blood glucose levels between 250 and 400 mg/dl were considered diabetic (Ozougwu, 2011).

### 2.10 Animal grouping

The animals were randomly grouped as follows with twelve albino (12) rats in each group:

- Group I: Non‐diabetic rats fed *D. rotundata* flour‐based diet.
- Group II: Diabetic control rats fed *D. rotundata*‐based diet.
- Group III: Diabetic rats fed *D. rotundata*‐based diet and administered metformin orally per day (14.2 mg/kg)
- Group IV: Diabetic rats fed unripe *M. paradisiaca‐*based diet

The diets were compounded as shown in Table 1. The diets and water were given to each group of animals and fed *ad libitum* for a period of 4 weeks.

**Table 1:** Compounded diets (g) for different groups of animals

Drugs/ingredients	Group I	Group II	Group III	Group IV
DF	57.60	—	—	—
A+DF	—	57.60	—	—
A+DF+M	—	—	57.60	—
A+UMP	—	—	—	57.60
Cellulose	6.00	6.00	6.00	6.00
Soybean	25.00	25.00	25.00	25.00
Soybeans oil	6.00	6.00	6.00	6.00
*Vitamin/Min mix	5.00	5.00	5.00	5.00
D‐methionine	0.40	0.40	0.40	0.40

A, alloxan; DF, *Dioscorea rotundata* (white yam flour); M, metformin; UMP, unripe *Musa paradisiaca* (unripe plantain).

*Mineral mix contained (g/ kg diet): CaCO3 (15.258), CoCl2.6H2O (0.001), ZnCl2 (0.001), CuSO4.5H2O (0.019), FeSO4.7H2O (1.078), MgSO4 (2.929), MnSO4.2H2O (0.178), KI (0.032), KH2PO4 (15.559) and NaCl (5.573), while the vitamin mix contained (g/kg diets): thiamine (0.02), riboflavin (0.03), pyridoxine (0.01), P‐aminobenzoic acid (0.20), myo‐inositol (2.00), biotin (0.001), menadione (0.01), ergocalciferol (0.4), choline‐HCl (2.0), and cellulose (3.31), α–tocopherol acetate (50 IU), retinal palmitate (4000IU), calcium pantothenate (0.0016) and folic acid (0.0002).

### 2.11 Food intake measurement and body weight of the rats

Food intakes were measured by subtracting the weight of serving dishes before and after meals. The body weights of the rats were measured in grams using weighing balance.

### 2.12 Blood sample collection and preparation of tissues supernatant

At the end of 4 weeks feeding period, the animals were humanely sacrificed under halothane euthanasia. The method described by Ogbu & Okechukwu (2001) was employed in blood collection and liver preparation.

### 2.13 Determination of biochemical parameters

Biochemical parameters evaluated in this study were as described for cholesterol (Trinder, 1969), triglyceride, and high‐density lipoprotein (Tietz, 1995), very low‐density, and low‐density lipoproteins (Friedewald, Levi, & Fredrickson, 1972); glycogen (Passoneau & Lauderdale, 1974), insulin (Gerbitz, 1950), hexokinase (Akinyosoye, Fawole, & Akinyanju, 1987), glucose‐6‐phosphatase (Swanson, 1950), and fructose‐1,6‐bisphosphatase (Gancedo & Gancedo, 1971).

### 2.14 Data analysis

The data were expressed as a mean ± standard error of mean (SEM) and were statistically analyzed by one‐way analysis of variance (ANOVA). Also for comparison of significance between groups, Duncan's test was used as a post hoc test according to the Statistical Package for the Social Sciences (SPSS) version 20.0, Chicago, IL, USA. A *p*‐value <.05 was considered statistically significant.

## 3 RESULTS

Proximate analyses of the compounded diets (Table 2) show there were significant difference (*p *<* *.05) between *M. paradisiaca* and *D. rotundata* flour‐based diets, with no significance difference (*p *>* *.05) in their moisture contents. The carbohydrate constituents, minerals and vitamins compositions of *M. paradisiaca‐based* diet (Table 2) were significantly higher (*p *<* *.05) than *D. rotundata* flour‐based diet. The levels of glucogenic amino acids in *M. paradisiaca*‐based diet (glycine, alanine, serine, proline, valine, threonine, aspartate, methionine, glutamate, histidine, arginine, tyrosine, and cysteine) were significantly higher (*p *<* *.05) than *D. rotundata* flour‐based diet (Table 2). Also, *M. paradisiaca‐based* diet demonstrated significance increase (*p *<* *.05) in the levels of monounsaturated and essential fatty acid, total phenol, flavonoid, ferric reducing power, nitric oxide, iron (II) chelation, dipheny‐1‐picrylhydrazyl, and hydroxyl radical (Table 2) when compared with *D. rotundata* flour‐based diet (Table 2).

**Table 2:** Macro‐ and micronutrient compositions of the compounded diets

Parameters (%)	Diet A (*Dioscorea rotundata* flour‐based diet)	Diet B (*Musa paradisiaca‐based* diet)
*Proximate analyses (%)*		
Ash	2.39 ± 0.15^a^	8.20 ± 0.12^b^
Lipid content	6.50 ± 0.12^a^	8.22 ± 0.12^b^
Crude fiber	8.39 ± 0.04^a^	16.62 ± 0.04^b^
Protein	16.89 ± 0.07^a^	19.49 ± 0.0^b^
Moisture content	1.68 ± 0.39^a^	1.87 ± 0.23^a^
Carbohydrate (by difference)	64.27 ± 0.35^a^	45.50 ± 0.42^b^
*Carbohydrate constituents*		
Arabinose (mg/100 g)	2.40 ± 0.01^a^	3.43 ± 0.12^b^
Fructose (mg/100 g)	2.60 ± 0.02^a^	4.40 ± 0.11^b^
IDF (%)	2.00^a^ ± 0.02^a^	4.28 ± 0.20^b^
SDF (%)	6.39 ± 0.05^a^	12.320.02^b^
*Minerals compositions (mg/100 g)*		
Mg	38.96 ± 0.32^a^	64.20 ± 0.02^b^
Zn	10.21 ± 0.02^a^	17.81 ± 0.06^b^
Se	0.21 ± 0.32^a^	1.40 ± 0.02^b^
Na	248.21 ± 0.02^a^	649.01 ± 0.34^b^
Ca	1.32 ± 0.02^a^	3.92 ± 0.03^b^
K	134.05 ± 0.02^a^	158.11 ± 0.02^b^
Fe	1.02 ± 0.42^a^	3.89 ± 0.02^b^
*Vitamin compositions (mg/100 g)*		
A	28.32 ± 0.02^a^	154.72 ± 0.22^b^
D	8.32 ± 0.09^a^	20.38 ± 0.02^b^
E	0.11 ± 0.08^a^	4.92 ± 0.34^b^
K	0.09 ± 0.02^a^	0.12 ± 0.02^b^
C	0.94 ± 0.32^a^	5.49 ± 0.05^b^
*Amino acids (g/100 g of protein)*		
Glycine* ^,^ †	1.89 ± 0.01^a^	2.99 ± 0.09^b^
Alanine* ^,^ †	3.24 ± 1.02^a^	7.99 ± 0.04^b^
Serine* ^,^ †	3.21 ± 0.12^a^	5.99 ± 0.22^b^
Proline* ^,^ †	3.82 ± 1.00^a^	8.21 ± 0.50^b^
Valine* ^,^ ‡	2.96 ± 1.02^a^	5.00 ± 0.05^b^
Threonine* ^,^ ‡	1.48 ± 0.22^a^	3.24 ± 0.05^b^
Isoleucine‡	2.40 ± 0.12^a^	4.42 ± 0.05^b^
Leucine‡	4.90 ± 1.02^a^	9.99 ± 0.05^b^
Aspartate* ^,^ †	5.20 ± 0.02^a^	5.89 ± 0.04^b^
Lysine‡	2.20 ± 0.02^a^	3.24 ± 0.02^b^
Methionine* ^,^ ‡	1.20 ± 0.02^a^	4.29 ± 0.02^b^
Glutamate* ^,^ †	12.20 ± 2.02^a^	18.20 ± 0.01^b^
Phenylalanine‡	3.26 ± 0.02^a^	4.20 ± 0.02^b^
Histidine* ^,^ ‡	2.22 ± 0.02^a^	4.52 ± 0.02^b^
Arginine* ^,^ ‡	3.27 ± 0.02^a^	3.94 ± 0.02^b^
Tyrosine* ^,^ †	1.01 ± 0.02^a^	2.92 ± 0.02^b^
Cysteine* ^,^ †	1.16 ± 0.02^a^	1.52 ± 0.02^b^
*Fatty acid composition (%)*		
Caprylic acid	0.05 ± 0.01^a^	0.09 ± 0.01^b^
Palmitic acid	10.98 ± 0.01^a^	18.98 ± 0.01^b^
Palmitoleic acid	0.35 ± 0.01^a^	1.12 ± 0.01^b^
Stearic acid	3.62 ± 0.01^a^	5.79 ± 0.01^b^
Oleic acids	18.32 ± 0.01^a^	25.89 ± 0.01^b^
Linoleic acid	40.89 ± 0.01^a^	44.60 ± 0.01^b^
Linolenic acid	1.20 ± 0.01^a^	2.96 ± 0.01^b^
Arachidic acid	1.10 ± 0.01^a^	1.14 ± 0.01^b^
Arachidonic acid	0.06 ± 0.01^a^	0.14 ± 0.01^b^
*Phenolic contents and in vitro antioxidant parameters of aqueous extract of the compounded diets (mg/g)*		
Total phenol (mg/g)	2.84 ± 0.02^a^	3.33 ± 0.01^b^
Total flavonoid (mg/g)	1.99 ± 0.20^a^	2.27 ± 0.30^b^
FRAP (%)	1.60 ± 0.09^a^	6.59 ± 0.03^b^
NO (%)	2.34 ± 0.03^a^	6.16 ± 0.08^b^
Fe^2+^ chelation (%)	34.23 ± 0.03^a^	57.76 ± 0.05^b^
DPPH (%)	23 01 ± 0.02^a^	41.231 ± 0.01^b^
OH (%)	6.04 ± 0.02^a^	9.52 ± 0.02^b^

Each value is a mean of three determinations ± SEM. Values with different superscripts across the rows are significantly different (*p *<* *.05).

FRAP, ferric reducing antioxidant power; NO, nitric oxide; OH, hydroxy radical; DPPH, 2,2‐diphenyl‐1‐picrylhydrazyl; IDF, insoluble dietary fiber; SDF, soluble dietary fiber.

*Glucogenic amino acids.

†Nonessential amino acids.

‡Essential amino acids.

In the first 2 weeks of the experiment there was no significance (*p *>* *.05) difference in food intake of diabetic control, diabetic rats maintained on metformin, and diabetic rats fed on *M. paradisiaca‐based* diet. But at 3rd and 4th weeks diabetic control rats showed significantly (*p *<* *.05) higher food intake (Table 3) when compared with the other groups. But there was reduction in food intake in diabetic rat's maintained on metformin and *M. paradisiaca‐*based diet.

**Table 3:** Compounded diets on food intake (g/day) of alloxan‐induced diabetic rats

Groups	Week 1	Week 2	Week 3	Week 4
Normal control	18.23 ± 1.22^a^	20.15 ± 2.22^a^	24.23 ± 2.10^a^	28.89 ± 2.16^a^
Diabetic control	25.24 ± 2.16^b^	30.34 ± 2.86^b^	36.69 ± 2.46^d^	46.29 ± 1.16^d^
Metformin	25.89.± 2.11^b^	29.56 ± 2.63^b^	34.50 ± 1.78^c^	41.23 ± 2.00^c^
*Musa paradisiaca‐*based diet	26.18 ± 2 .20^b^	29.68 ± 2.32^b^	32.34 ± 1.65^b^	38.45 ± 1.89^b^

Each value is a mean of 11 determinations ± SEM. Values with different superscripts along the column are significantly different (*p *<* *.05).

**Figure 1:**
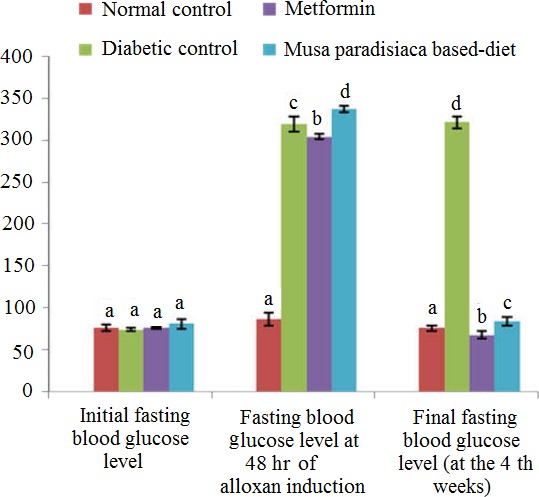
Fasting blood glucose levels (mg/dl) of alloxan‐induced diabetic rats fed *Musa paradisiaca‐based* diet. Each value is a mean of 11 determinations ± SEM, Values with different superscripts are significantly different (*p *<* *.05)

At the end of 4 weeks of experimentation, the normal control rat was found to increase in final body weight and percentage weight gain (Table 4). The diabetic rats maintained on *M. paradisiaca‐based* diet showed significant increase (*p *<* *.05) in final body weight and percentage weight gain when compared with diabetic control and metformin‐treated groups. Also, the fasting blood glucose levels (Figure 1) were significantly higher (*p *<* *.05) in the diabetic control rats when compared with diabetic rats fed *M. paradisiaca‐*based diet for a period of 4th weeks.

**Table 4:** Compounded diets on the body weight changes (g) of alloxan‐induced diabetic rats

Groups	Initial body weight	Final body weight	Percentage weight gain/loss
Normal control	150.21 ± 4.62^a^	177.45 ± 2.45^a^	18.13 ± 2.89^a^
Diabetic control	148.12 ± 6.21^a^	99.75 ± `1.77^d^	−32.66 ± 1.66^d^
Metformin	150.23 ± 4.21^a^	119.73 ± 2.11^c^	−20.30 ± 1.07^c^
*Musa paradisiaca‐*based diet	146.32 ± 5.60^a^	135.82 ± 1.02^b^	−7.18 ± 1.65^b^

Each value is a mean of 11 determinations ± SEM. Values with different superscripts along the column are significantly different (*p *<* *.05).

The levels of serum cholesterol, triglycerides (TG), very low‐density lipoprotein (VLDL), and low‐density lipoprotein (LDL) (Table 5) were significantly higher (*p *<* *.05) in diabetic control rat when compared with diabetic rats placed on *M. paradisiaca‐*based diet. However, the serum levels of HDL were significantly reduced (*p *<* *.05) in diabetic control rats when compared with diabetic rats maintained on *M. paradisiaca‐*based diet.

**Table 5:** Serum lipid profile of alloxan‐induced diabetic rats fed *Musa paradisiaca‐based* diet for 4 weeks

Groups	Cholesterol (mmol/L)	TG (mmol/L)	HDL (mmol/L)	VLDL (mmol/L)	LDL (mmol/L)
Normal control	75.48 ± 0.25^a^	43.71 ± 0.09^a^	34.18 ± 0.04^a^	19.87 ± 0.19^a^	21.19 ± 0.03^a^
Diabetic control	117.55 ± 0.13^d^	67.03 ± 0.01^c^	17.05 ± 0.01^c^	30.47 ± 0.02^b^	69.94 ± 0.12^d^
Metformin	77.91 ± 0.05^b^	43.52 ± 0.12^a^	34.70 ± 0.62^a^	19.78 ± 0.21^a^	22.76 ± 0.10^b^
*M. paradisiaca‐based* diet (MD)	81.39 ± 0.09^c^	44.51 ± 0.40^b^	33.85 ± 0.04^b^	19.93 ± 0.20^a^	27.11 ± 0.02^c^

Each value is a mean of 11 determinations ± SEM. Values with different superscripts along the column are significantly different (*p *<* *.05).

HDL, high‐density lipoprotein; VLDL, very low‐density lipoprotein; LDL, low‐density lipoprotein.

**Figure 2:**
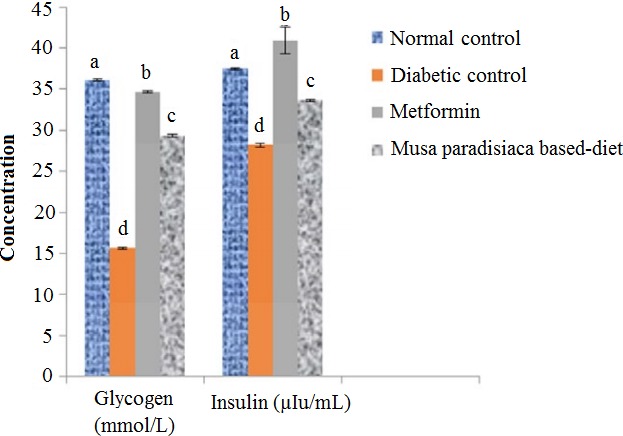
Hepatic glycogen (mmol/l) and serum insulin concentrations (μIu/ml) of alloxan‐induced diabetic rats fed *Musa paradisiaca‐based* diet for 4 weeks. Each value is a mean of 11 determinations ± SEM, Values with different superscripts are significantly different (*p *<* *.05)

The hepatic glycogen concentration and serum insulin levels (Figure 2) were significantly reduced (*p *<* *.05) in diabetic control rats, compared with diabetic rats fed on *M. paradisiaca‐*based diet. However, at the end of 4 weeks feeding trial, there was significant increase (*p *<* *.05) in hepatic glucagon and insulin concentrations of diabetic rats placed on *M. paradisiaca‐based* diet.

**Figure 3:**
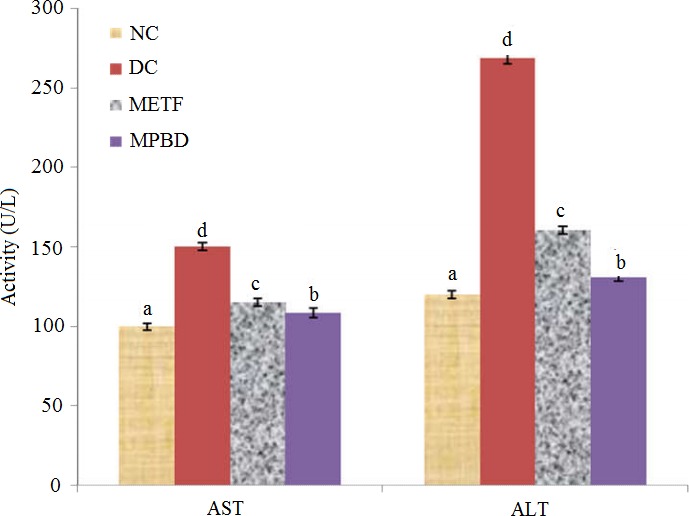
Activities of AST and ALT in liver of alloxan‐induced diabetic rats fed *Musa paradisiaca‐*based diet for 4 weeks. Each value is a mean of 11 determinations ± SEM, Values with different superscripts are significantly different (*p *<* *.05). NC: normal control, DC: diabetic control, METF: metformin, MPBD:* Musa paradisiaca‐*based diet, AST: aspartate aminotransferase and ALT: alanine aminotransferase

The activities of glucose‐6‐phosphatase and fructose 1, 6 bisphosphatase (Table 6) were significantly increased (*p *<* *.05) in diabetic control rats with significance decrease (*p *<* *.05) in hexokinase activity. At the end of feeding trial, the activities of glucose‐6‐phosphatase and fructose 1, 6‐ bisphosphatase were significantly decreased (*p *<* *.05) while that of the hexokinase activity was significantly increased (*p *<* *.05) in diabetic rats placed on *M. paradisiaca‐*based diet. Furthermore, the activities of liver AST and ALT (Figure 3) were significantly higher (*p *<* *.05) in diabetic control rat when compared with diabetic rats placed on *M. paradisiaca‐*based diet and others groups.

**Table 6:** Some hepatic carbohydrate metabolism enzymes activities in tissues of alloxan‐induced diabetic rats fed *Musa paradisiaca‐based* diet for 4 weeks

Groups	Gluocose‐6‐phosphatase (μm of glucose phosporylated/hr/mg/dl)	Hexokinase (μm of Pi liberated/hr/mg/dl)	Fructose 1,6 diphosphatase (μm of Pi liberated/hr/mg/dl)
Normal control	845.36 ± 2.32^a^	156.10 ± 0.17^a^	400.18 ± 0.35^a^
Diabetic control	1357.2 ± 6.12^d^	100.48 ± 0.37^d^	770.41 ± 8.14^d^
Metformin	904.24 ± 2.94^b^	154.71 ± 0.08^b^	431.17 ± 0.41^b^
*M. paradisiaca*‐based diet	971.98 ± 1.61^c^	140.29 ± 0.11^c^	445.11 ± 0.06^c^

Each value is a mean of 11 determinations ± SEM. Values with different superscripts along the column are significantly different (*p *<* *.05).

## 4 DISCUSSION

Diabetes mellitus has been reported as a chief killer disease all over the world, characterized with hyperglycemia and dyslipidemia. Several drugs have been used for its management but are characterized with serious side effects (Ogbonnia, Odimegwu, & Enwuru, 2010). This lead to an increase in demand for natural products, series of herbs have been reported to be useful as antihyperglycemia but they are distinguished with side effects (Okoli, Agbe, Ohaju‐Obodo, & Mensah, 2007). The first line of management often advocated is dietary approach (Johnson, Isaac, Michael, Akintayo, & Samuel, 2013), whereby food performs the function as medicine.

The proximate analyses of *M. paradisiaca* revealed the presence of ash (Table 2), an indicator of rich mineral compositions. Wali, Jogana, Zarummai, & Saidu (2011) reported that antioxidant minerals (such as zinc, selenium, etc.) (Table 2) are highly useful in the regeneration of damaged beta cells of pancreas, also, the presence of sodium and potassium in the diet may ameliorate dehydration in diabetes mellitus patient (Wali, Jogana, Zarummai, & Saidu, 2011). The availability of linoleic, linolenic, and arachidonic acids in ether extract of *M. paradisiaca‐*based diet (Table 2) may also be useful in regeneration of damaged beta cells and in speeding up wound healing processes in diabetes mellitus patient; this was coupled with the presence of caprylic acid (Table 2), which has the beneficial effect on wound healing by penetrating the membrane due to its short chain (Yin, Bai, & Jing, 2014). The dietary fibers may delay the digestion and conversion of starch to glucose, and therefore retard the absorption of glucose from the gastrointestinal tract. This was coupled with the presence of soluble dietary fiber, arabinose, and fructose in both the sample and its compounded diet (Table 2) which delaying gastric emptying, inhibiting glucagon secretion, and stimulating insulin secretion in diabetic mellitus animals (Li & Mandeep, 2010).

Moreover, diabetes mellitus is characterized with gastrointestinal dysfunction due to hyperglycemia (Rodrigues & Molta, 2012). This may also be ameliorated by the presence of insoluble dietary fiber in the *M. paradisiaca‐*based diet (Table 2). Muscle wasting is one of the commonest symptoms of diabetes mellitus but the presence of protein and amino acids in the sample and its compounded (Table 2) may be useful in ameliorating this effect. The presence of glycine, arginine, cysteine, and methionine has been reported by Naik (2011) in detoxification of reactive oxygen species making them useful in regeneration of damage beta cells. The availability of glucogenic amino acids such as glycine, alanine, serine, proline, valine, threonine, aspartate, methionine, glutamate, histidine, arginine, tyrosine, and cysteine may be helpful in regenerating the wasted muscle (Karri & Srinivasan, 2013). Furthermore, one of the main causes of diabetes mellitus is the accumulation of free radicals, the presence of antioxidant vitamins (such as vitamins A, C, and E) (Table 2) in *M. paradisiaca‐*based diet, which are free radical scavengers and immune system boosters has also been reported by Wali, Saidu, Ladan, Bilbis, and Ibrahim (2013) in the regeneration of damaged free radicals. This was supported by the level of total phenol, flavonoid, and *in vitro* antioxidant parameters (DPPH, iron chelation, nitric oxide among others) (Sivajothi, Dey, Jaykar, & Rajkapoor, 2010) in the sample and its compounded diet (Table 2).

Diabetes mellitus has been characterized with polyphagia, which may be responsible for observed increase in food intake (Table 3). This is important for the animals to compensate for loss of body weight (Table 4) and fluid (Irshaid, Mansi, & Aburjai, 2010). But at the end of the feeding trial, diabetic rats fed on *M. paradisiaca‐*based diet showed reduction in food intake. This might be attributed to normoglycemic activities of *M. paradisiaca‐*based diet.

In this study, the significant decrease in the weights of the diabetic control rats (Table 4) may be attributed to degeneration of adipocytes and muscle tissues, which may be due to catabolism of proteins and fats in the body of diabetic mellitus rats (Esonu, Emenalom, Udedibie, Herbert, Ekpor, Okoli, & Ihukwumere, 2001). The increase in body weight of the rats at the end of feeding trail may be associated with the glucogenic amino acids and fructose in the diet among others. The intraperitoneal injection of rats with alloxan monohydrate significantly increases the blood glucose due to destruction of insulin producing organ (beta cells of pancreas), thereby causing hyperglycemia (Figure 1), in the absence of insulin, the tissues (adipose tissue, etc.) are unable to use glucose (Sharma, Kumar, Patel, & Hugar, 2010). But at the end of the feeding trial, the *M. paradisiaca‐*based diet was able to normalize the hyperglycemia to normolglycemia, an indication that the diet may act by stimulating insulin secretion and promotes utilization of glucose by peripheral tissue probably due to fiber, antioxidants among others (Suganya, Narmadha, Gopalakrishnan, & Devaki, 2012).

Alterations in serum lipid profiles (Table 5) are known in diabetics, probably due to increase in the mobilization of free fatty acids from the peripheral depots, as insulin inhibits the hormones lipase (Radhika, Smila, & Muthezhilan, 2011). The diabetic rats treated with *M. paradisiaca‐*based diet reversed this abnormality with increase in high‐density lipoprotein concentration, probably by enhancing the insulin secretion.

Glycogen (Figure 2) is the storage form of glucose in the liver, reflection of insulin concentration (Naik, 2011) (Figure 2). The observed decreased in glycogen concentration in the diabetic rats may be attributed to reduction in insulin levels. Whereas, at the end of feeding trial, the concentrations of these two parameters were elevated, this may be due to increase in insulin sensitivity, secretion, and enhances glycogen synthase (Abd El‐Rasek & Hassan, 2011). This may be one of the reasons responsible for normal glycemic potential of *M. paradisiaca‐*based diet.

Liver is an organ involved in glucose homeostasis, the main site of glycolysis and gluconeogenesis. There was impairment of hexokinase enzyme (key enzyme in glycolysis) activity in diabetes mellitus probably due to deficiency of insulin. Likewise, glucose‐6‐phosphatase and fructose‐1, 6‐bisphosphatase activities (key gluconeogenesis enzymes) (Table 6) were significantly increased in diabetic control rats, due to insulin deficiency (Ragavan & Krishnakumari, 2006). These abnormalities were restored after feeding the diabetic rats with *M. paradisiaca‐*based diet due to increase in insulin secretion and its sensitivity by the diet.

Aspartate aminotransferase and alanine aminotransferase are normally used to assess liver toxicity, changes in serum enzymes activities are directly associated with alteration in the physiological functions of aspartate aminotransferase and alanine aminotransferase in alloxan‐induced diabetic animals as reported by Asayama, Nakane, Uchida, Hayashibe, Dobashi, & Nakazawa (1994). Kazeem, Akanji, Yakubu, & Ashafa (2013) reported that elevated activities of transaminases under insulin deficiency could be responsible for increased gluconeogenesis and ketogenesis during diabetes state, which was observed in this study. Also, the increased activities of transaminases are pointer of hepatic damage. Conversely, feeding of diabetic rats with *M. paradisiaca‐*based diet caused reduction in the activities of the two enzymes and probably alleviates liver damage.

## 5 CONCLUSION

It can be concluded from this study that *M. paradisiaca‐based* diet has antihyperglycemic and antidyslipidemia potential and might be useful for diabetic mellitus patients, probably due to its fiber, soluble dietary fiber, fructose, amino acids contents, and antioxidant potentials among others in *M. paradisiaca‐*based diet.

## CONFLICT OF INTEREST

The authors have no conflicts of interest.

## ETHICAL APPROVAL

This research was approved by the ethical committee of the University of Ilorin, Ilorin, Kwara State, Nigeria.
